# Contraception and Abortion Care for People Living With HIV: A Clinical Guide for Reproductive Health Practitioners

**DOI:** 10.1111/jmwh.13575

**Published:** 2023-10-30

**Authors:** Lanbo Yang, Rebecca H. Allen, Mary Catherine Cambou, Karin Nielsen-Saines, Benjamin P. Brown

**Affiliations:** 1 Department of Obstetrics and Gynecology, Tulane University School of Medicine, New Orleans, Louisiana; 2 Department of Obstetrics and Gynecology, Women and Infants Hospital/Warren Alpert Medical School of Brown University, Providence, Rhode Island; 3 Division of Infectious Diseases, Department of Medicine, UCLA David Geffen School of Medicine, Los Angeles, California; 4 Division of Pediatric Infectious Diseases, Department of Pediatrics, UCLA David Geffen School of Medicine, Los Angeles, California

**Keywords:** contraception, HIV, birth control, abortion, drug interactions

## Abstract

People capable of pregnancy are disproportionately affected by HIV. Family planning needs and services are often unmet in this population, and clinical care guidelines regarding contraceptive options and abortion care are not well elucidated Individuals living with HIV often face unique barriers in accessing contraception and abortion services due to internalized stigma, medically complex care (eg. drug–drug interactions, adverse effects of antiretroviral therapy), and distrust of health care providers. There is also a lack of clarity among reproductive health, primary, and infectious disease care providers on best-practice contraceptive counseling and contraceptive care for individuals living with HIV, given limited opportunities to enhance expertise in reproductive infectious disease. In this review, we summarize existing and updated evidence and clinical considerations regarding contraceptive counseling and abortion care in this population.

## INTRODUCTION

### Reproductive-Age People Living With HIV

Worldwide, people capable of pregnancy are disproportionately affected by HIV, and vertical transmission is the global leading cause of pediatric HIV.^1^ There are 38.4 million people living with HIV worldwide, 54% of whom identify as female and of reproductive age.^2^ In sub-Saharan Africa, female-identifying and reproductive-age individuals comprise 63% of new HIV infections in 2021. Worldwide, HIV affects cisgender women the most: in 2016, there were an estimated 2.4 million adolescent cisgender adolescent girls and young women living with HIV, who comprise 61% of all young people living with HIV.^3^ Since the advent of combination antiretroviral, therapy (cART), recent studies in the United States and abroad have demonstrated that pregnancy and live birth rates among women living with HIV are now similar and comparable to women without HIV.^4,5^ Changing fertility intentions, reduced fear of vertical transmission, and increasingly positive attitudes that decrease societal stigma may have contributed to this increase in live birth and pregnancy rates among people living with HIV, which were previously low in the era before cART.^5,6^ In this review, we report data based on gender criteria from the original studies, which may only include a sample of one gendered population (eg, cisgender women), but we recognize that these extrapolations are gendered and may not be applicable to gender diverse populations. We acknowledge that there are people of all genders who are nonetheless capable of pregnancy and whose care may therefore be affected by our recommendations. Additionally, we acknowledge that the term *family planning* used in this review encompasses a broad and inclusive understanding of sexual and reproductive health needs and desires.

### Unmet Need for Abortion and Contraception Access

Contraception and abortion are critical aspects of reproductive health care for all people, including those living with HIV. Consequently, professionals who provide contraception and abortion care can expect to see patients who are living with HIV. About half of all pregnancies in the United States are unintended,^7^ and this rate has been reported to be around 78% among women living with HIV in the United States.^8^ Recent studies from the United States and South Africa have suggested that social and structural barriers play a key role in limiting access to prenatal care, viral load monitoring, and cART treatment, which in turn may increase the likelihood of unsuppressed viremia at the time of birth and subsequent risk of vertical transmission.^9,10^ In the United States, the rate of long-acting reversible contraception use among women living with HIV is lower compared with women without HIV.^4,11,12^ Moreover this trend is observed in both resource-constrained as well as in resource-abundant settings, highlighting a potential unmet need for family planning services globally. ^13–16^ Clinicians who provide family planning care for people capable of pregnancy should be equipped to provide contraception and abortion care to people living with HIV. This review serves as a resource for reproductive health care practitioners faced with clinical scenarios at the intersection of HIV and family planning. From a practitioner’s perspective, enhancing familiarity and comfort with prescribing contraceptive options is equally important to addressing cultural and social stigma, as this may change practice patterns. Shared decision-making and motivational interviewing are important strategies to address issues and empower patients in the counseling process.^17^

### CONTRACEPTIVE OPTIONS FOR PEOPLE LIVING WITH HIV

It is widely acknowledged that barrier methods—namely, penile and internal condoms—play a pivotal role in both reducing HIV transmission and increasing contraceptive efficacy. As counseling on and knowledge of barrier methods as a method of preventing HIV transmission are both widespread, for this review we focus primarily on clinical considerations for nonbarrier methods of contraception. Due to shared metabolic pathways through the cytochrome (CYP) 450 enzymes in the liver, hormonal contraception and cART may have drug–drug interactions that decrease contraceptive efficacy and, in certain cases, may lead to altered levels of antiretroviral drugs. Antiretroviral therapy (ART) may induce or inhibit CYP450 enzymes, thus altering hormone metabolism and glucuronidation.^18^ Studies investigating the clinical relevance of these drug–drug interactions contain small sample sizes and focus mainly on pharmacokinetics and pharmacodynamics. Therefore, it is difficult to ascertain clinical applicability, which remains, at this point, largely theoretical. Each clinician should take an individualized approach to contraception counseling among patients with HIV. Although this review highlights important themes from recent studies on interactions between contraception or abortion therapies (eg, medication abortion with misoprostol and mifepristone or procedural abortion) and HIV treatment, we also recommend regularly reviewing documents detailing expert opinions and specific drug–drug interactions from the US Department of Health and Human Services Panel on Antiretroviral Guidelines for Adults and Adolescents^19^ as well as the *US Medical Eligibility Criteria for Contraceptive Use* from the Centers of Disease Control and Prevention.^20^ A third resource that may be particularly helpful to clinicians is the Liverpool University HIV Drug Interactions website, which reports drug–drug interactions with antiretroviral medications.^21^ Table 1 provides a summary of the key recommendations.

### Copper and Levonorgestrel-Releasing Intrauterine Devices

One key concern clinicians may have regarding intrauterine devices (IUDs) and HIV is the potential for pelvic infection. Although concerns were raised in the 1970s about an association between a specific US-marketed IUD and an increased risk of salpingitis, the data on which these concerns were based are not definitive. More importantly, no currently available IUD is associated with an increased risk of pelvic inflammatory disease.^22,23^ This remains true even for people living with HIV, as demonstrated by a recent study from a cohort of 156 women living with HIV that did not demonstrate an association between HIV infection and IUD-related complications.^24^ With regard to drug–drug interactions, it is thought that levonorgestrel IUDs exert their effects pre-dominantly through local, not systemic, absorption; thus it is unlikely that ART inhibits their contraceptive efficacy.^25,26^ Likewise, ART effectiveness is unlikely to be affected by levonorgestrel IUDs: in laboratory settings, levonorgestrel-releasing IUDs did not increase genital HIV RNA shedding or inflammatory markers.^27^ Clinically, there are no significant differences in HIV disease status between users of the levonorgestrel and copper IUDs: a recent randomized, non-inferiority trial from South Africa of more than 200 women living with HIV demonstrated that levonorgestrel IUDs were not associated with changes in genital tract or plasma HIV viral load when compared with copper IUDs.^28^ Furthermore, in a study using the copper IUD as the reference standard for comparing contraceptive efficacy and IUD-associated complications, levonorgestrel-containing IUDs had similarly low levels of contraceptive failure and IUD complications such as expulsion. Copper IUDs are assumed to not confer risk of increased HIV acquisition or interact with HIV treatment drugs because they do not release hormones. A systemic review from 2020 found no association between the copper IUD and HIV infection.^29^

### Etonogestrel/Levonorgestrel Implants

In the United States, the only subdermal contraceptive implant currently available contains etonogestrel. Globally, however, both etonogestrel and levonorgestrel implants are available, and US clinicians may care for patients using either type of implant. Progestin-based implants are generally effective among individuals living with HIV, and given their very high contraceptive effectiveness at baseline, even a relative decrease due to drug–drug interactions may leave patients with meaningful protection against pregnancy. However, implants may have decreased clinical effectiveness when combined with certain ART drugs, such as efavirenz. Pharmacokinetic and clinical studies have consistently reported efavirenz is associated with decreased levels of progestins by as much as 40% to 82%, leading to increased rates of contraceptive failure and unintended pregnancies.^30–35^ In one study, one-third of dual users of efavirenz and a levonorgestrel implant had lowered progestin concentrations below the minimum threshold required to suppress ovulation.^34^ This combined detrimental effect on contraceptive efficacy may be further worsened by patient genetic factors, such as carrying the CYP2B6 single-nucleotide polymorphism mutation.^36^ This finding appears to be limited to efavirenz-containing antiviral therapies, as it was not observed with other ART regimens such as ritonavir/atazanavir, ritonavir/lopinavir, or nevirapine.^31,37^ Limited data from cohort studies have demonstrated significantly increased risk of contraceptive failure with levonorgestrel and etonogestrel implants for efavirenz users, which prompted certain countries such as South Africa to recommend against the use of subdermal implants for efavirenz users in 2014.^38–40^ New and emerging data from larger population-based cohorts suggest that contraceptive efficacy of the subdermal implant is not diminished with concurrent efavirenz use and may even be as effective as depot-medroxyprogesterone acetate (DMPA).^40^ Additionally, these drug–drug interactions are not observed at later time intervals of use of progestin implants, suggesting that shortening of implant use or replacing the implant sooner for efavirenz users may not be warranted.^33^ Currently, the use of subdermal implants for users of efavirenz remains an area of active research, and if the subdermal implant is a patient’s preferred method, consultation with an infectious disease specialist may be preferable to explore other first-line ART regimens such as dolutegravir-based therapies. A recent study in Botswana has shown that, unlike efavirenz, dolutegravir increases etonogestrel concentrations and is unlikely to have a detrimental effect on contraceptive efficacy.^41^ With regard to other ART agents, patients using darunavir/ritonavir exhibit decreased progestin levels, as with combined oral contraceptives (COCs), compromising contraceptive efficacy; alternative and additional contraception may be sought in these patients after consultation with infectious disease specialists.^42^ In summary, although etonogestrel and levonorgestrel implants appear to retain meaningful contraceptive efficacy even among people on CYP450-inducing ART medications, to optimize contraceptive effectiveness, the use of first-line ART regimens that do not include efavirenz, darunavir, or ritonavir is preferred when a progestin implant is chosen as the preferred contraceptive method.^33^

### Injectable Contraceptives: DMPA

Studies to date suggest that drug–drug interactions between DMPA and ART are generally not clinically significant. In terms of ART drug levels, DMPA did not change drug concentrations of efavirenz, nelfinavir, and lopinavir/ritonavir.^43,44^ Evidence suggests that efavirenz and nevirapine have no effect on contraceptive efficacy and do not affect ART levels or HIV disease progression.^40,43,45–47^ Certain ART drugs, such as lopinavir/ritonavir, may actually increase DMPA levels, although this is unlikely to be dangerous.^44^ In terms of contraceptive efficacy of injectable contraceptives with new and emerging ART regimens, HPTN 084 was a phase 3 trial from 2017 to 2020 that investigated cabotegravir, the first injectable form of ART approved by the US Food and Drug Administration last year, for pre-exposure prophylaxis (PrEP) in cisgender women within the age range of 18 to 45 who were using a form of long-acting hormonal contraception. Among 1184 participants in the cabotegravir group, of whom 1009 were using DMPA and 175 were using norethisterone enanthate (an intramuscular injectable progestin-only contraceptive method similar to DMPA that is unavailable in the United States but used widely in various countries in Europe, South America, and Africa), 29 pregnancies occurred (1.5 per 1000 person-years).^48^

However, adverse effects unrelated to contraceptive or HIV treatment may be of concern with tenofovir disoproxil fumarate (TDF), a commonly used nucleoside reverse transcriptase inhibitor (NRTI) and first-line medication. A 2022 randomized controlled trial conducted in Uganda and including 265 women living with HIV raised concerns of accelerated bone loss with concurrent use of intramuscular DMPA and TDF.^49^ Although both intramuscular DMPA and TDF have been shown to be independently associated with decreased bone density, this was the first prospective study to demonstrate their combined deleterious effect, which is almost a 2-fold decrease in bone mineral density loss as opposed to women on either DMPA or TDF. This trend was observed at different CD4 cell counts and unlikely to be related to uncontrolled HIV infection. It is unclear what the long-term consequences of this bone loss may be, as fracture risk data are lacking, and it is difficult to ascertain fracture risk in matched individuals with and without HIV.

### Combined Hormonal and Progestin-Only Oral Contraceptives

COCs generally do not have clinically significant effects on ART drug levels, as demonstrated by studies of COCs and various ART drugs such as efavirenz, nevirapine, rilpivirine, and dolutegravir,^50–54^ although one study reported subtherapeutic efavirenz concentration levels in 3 of 16 patients.^53^ Furthermore, in a subanalysis of HPTN 077, a phase 2a trial that investigated the use of cabotegravir for PrEP in the general population, cabotegravir levels decreased in 18 cisgender women on COCs compared with those who were not on contraception.^55^

COCs and progestin-only contraceptive pills may exhibit decreased hormone levels with certain ART regimens, although their clinical significance is not yet clear. Pharmacokinetic changes such as decreased ethinyl estradiol levels have been previously observed in women living with HIV using COCs while on a nonnucleoside reverse transcriptase inhibitor (NNRTI) or protease inhibitor.^56,57^ For patients who are taking darunavir/ritonavir and on COCs, additional contraception may be required due to inadequate hormone levels.^58^ Progestin levels may also be affected by ART medications: although a prospective study in 2019, which followed 15 women on ritonavir-boosted protease inhibitors, did not show any difference in ethinyl estradiol concentrations, the authors did find increased levels of levonorgestrel.^59^ Likewise, an earlier prospective, nonrandomized trial suggested that norethisterone-based progestin-only contraceptives exhibit increased drug levels when co-administered with ritonavir-boosted protease inhibitors.^60^ However, not all ART medications demonstrate alterations in contraceptive hormone pharmacokinetics: a recent phase 1 trial found that the pharmacokinetics of COCs (levonorgestrel/ethinyl estradiol) were not altered with islatravir (a novel NRTI) and did not require dose adjustment.^61^

Another drug–drug interaction of clinical concern is hyperkalemia when drospirenone-containing contraceptives are used along with cobicistat-containing ART regimens.^58^ Currently drospirenone-containing contraceptives are generally contraindicated in users of cobicistat-boosted protease inhibitors when an alternative or additional contraceptive method is available.^19^

In terms of the combined hormonal vaginal contraceptive rings, a study in 2019, including 84 women living with HIV, showed that efavirenz decreased etonogestrel levels by 79% and ethinyl estradiol levels by 59%, whereas ritonavir-boosted atazanavir (a protease inhibitor) increased etonogestrel levels by 71% and lowered ethinyl estradiol concentrations.^62^ A secondary analysis of this cohort of women showed that etonogestrel levels were decreased by 93% in a certain subset of women on efavirenz with the slow-metabolizing CYP genotype (CYP2B6), suggesting that contraceptive counseling with these individuals would be optimized in close collaboration with the patient’s infectious disease/HIV care provider.^63^ With regard to genotype testing and the goal of boosting an antiretroviral from an infectious disease perspective, it is recommended that individuals initiating ART have viral genotypic susceptibility testing performed. Susceptibility testing detects the presence of potential viral genetic mutations associated with specific antiretrovirals within diverse classes of drugs, including NNRTIs, NRTIs, protease inhibitors, and integrase inhibitors. Pharmacologic and pharmacokinetic data are available regarding potential CYP pathways used for metabolism of each of these drugs. Typically, protease inhibitors are antiretrovirals that require hepatic metabolism and most frequently use CYP pathways that compete with those of other drugs. Therefore, although CYP genotypes are not routinely tested per se, susceptibility testing enables identification of ART, which will be effective in reducing viremia. The practitioner can then decide to use effective ART that may not require metabolic pathways that potentially interact with other drugs. However, knowledge of CYP pathway metabolism is a priori and does not require genotypic testing. Some antiretrovirals require the use of a secondary medication that increases drug levels of the main antiretroviral drug to maximize therapeutic levels, which is termed *boosting*. Boosted atazanavir, for example, refers to the use of atazanavir with a second agent, ritonavir, which increases atazanavir levels. Although ritonavir is another protease inhibitor with ART activity, in this case it is used primarily to increase therapeutic levels of atazanavir. Some antiretrovirals can be given in boosted or unboosted formulations. Knowledge of ART drug metabolism is important to predict drug–drug interactions.

Unboosted atazanavir may also increase levels of ethinyl estradiol because of drug–drug interactions that may lead to increased adverse events; however, this has yet to be studied.^19^ Other protease inhibitors without boosted ritonavir include fosamprenavir and nelfinavir where theoretical concerns have also been raised given that drug–drug interactions with hormonal contraception may lower the ART drug levels; however, this has not been studied. These concerns with unboosted protease inhibitors also apply to DMPA and progestin-only contraceptives. With fosamprenavir, ART drug levels may decrease with any hormonal contraceptive due to CYP3A4 pathways, and this interaction may be less with progestin-only contraceptives. When considering DMPA and progestin-only contraceptives with nelfinavir, contraceptive efficacy and ART drug levels may be diminished as well.^19^ These studies suggest that further research needs to investigate the contraceptive efficacy of intravaginal ring for patients on protease inhibitors. In terms of the hormonal patch, the data are insufficient to draw clinical conclusions about contraceptive efficacy: one study of 8 women taking Iopinavir/ritonavir found decreased ethinyl estradiol levels and increased progestin levels with continually suppressed ovulation.^64^ In terms of ART drug levels, one study found lopinavir/ritonavir concentrations were decreased by 19% in 8 women with concurrent hormonal patch use (ethinyl estradiol and norelgestromin); however, there are no clinical studies to suggest clinical significance on HIV disease progression.^64^

### ABORTION CARE FOR PEOPLE LIVING WITH HIV

#### Medication Abortion

Data are limited regarding complications and outcomes of medication abortion in women living with HIV. To date, the largest known study of efficacy and safety of medication abortion in this population was a small case series in Ukraine, which included 68 women living with HIV who were not on ART.^65^ In this study, the medication abortion regimen was mifepristone 250 mg orally in the hospital followed by misoprostol 400 mcg sublingually 36 to 48 hours later.^66^ Medication abortion was highly effective (96%), with low rates of complications. Among the 68 women, there was one incomplete abortion, one case of heavy bleeding, and one case of ongoing pregnancy with no cases of serious postabortion infections. In another study in Uganda, comparing complications following induced abortion between women with self-reported HIV and those without HIV, there was no difference in the rates of women who returned to the hospital seeking care for management of incomplete abortion. However, in this study, the authors did not specify the mode or modes of abortion (medical or procedural) used during the study period.^67^ Other than the study in Ukraine, a Cochrane review from 2018 found no other studies pertaining to the safety of medication abortion among individuals living with HIV, suggesting a knowledge gap and a need for further research.^66^ To our knowledge, there are no studies that explore drug–drug interactions between misoprostol or mifepristone with antiretroviral treatment.

It is worthwhile to note that medical complications of HIV disease—particularly, anemia and thrombocytopenia—may affect whether an individual is a safe candidate for medication abortion. However, these complications typically depend on viral suppression and active treatment. If a patient has stable, undetectable viral loads (<50 copies/mL) and is actively taking antiretrovirals, health care providers do not need to exercise any extra precaution for cytopenia for people living with HIV.^68–70^ In terms of drug–drug interactions between medication used for abortion and antiretroviral therapies, there is currently no known drug–drug interaction between misoprostol and any existing HIV antiretroviral drug. As for mifepristone, there are potential drug–drug interactions that have yet to be studied but are unlikely to be clinically significant. Mifepristone is metabolized by a specific enzyme in the liver (CYP3A4), and thus HIV antiretroviral drugs that induce this enzyme (eg, efavirenz) may lead to decreased systemic concentrations of mifepristone, whereas ART drugs that inhibit this enzyme (eg, atazanavir) may lead to slower metabolization of mifepristone and therefore increased systemic concentrations. Patients who are taking an ART antiretroviral drug that is a CYP3A4 inducer should be monitored for clinical efficacy of mifepristone. A summary of these drug-drug interactions can be found using the Liverpool University HIV Drug Interactions resource.^21^

#### Procedural Abortion

Similar to medication abortion, the data regarding outcomes and complications following procedural abortion are limited. The studies that do exist, most of which are small case-control and cohort studies, generally show that there is no increased risk of postabortion infectious complications; however, there may be an increased risk of other general complications of procedural abortion. Due to the variability of the proportions of patients living with HIV who were actively on antiretroviral treatment in these studies, it is difficult to attribute increased risk to HIV disease status. Biologically speaking, a chronic proinflammatory state may delay wound healing in advanced HIV states; however; if the viremia is undetectable, HIV disease generally should not affect recovery from procedures. In a study of general perinatal and gynecologic surgical procedures among 173 women living with HIV in Germany, the rate of postoperative complications following 72 dilation and curettage procedures in women living with HIV was greater (9.7%) than in women without HIV (1.4%); however, this difference failed to reach statistical significance (*P* = .063).^71^ Although this difference was not statistically significant, the small sample size and paucity of outcome events (one complication in 71 women without HIV and 7 complications in 72 women with HIV) weaken any conclusions that could be drawn or any associations that were reported after a curettage procedure (odds ratio, 3.8; 95% CI, 0.76–55; *P* = .1) The complications following dilation and curettage procedures in this study were not specified. A subsequent cohort study by Stuart and colleagues reviewed data from 71 women living with HIV in Dallas who underwent dilation and curettage for the management of spontaneous or induced abortion and demonstrated significantly higher rates of any complication (14% in the HIV group and 4% in the control group), which were defined as hemorrhagic, infectious, operative, anesthesia-related complications (3% in the HIV group vs 0 cases in the control group), and retained placenta requiring a repeat procedure (4% in the HIV group vs 0 cases in the control group).^72^ Of note, only 38% of this cohort were taking antiretroviral medications, and 25% met criteria for advanced disease and diagnosis of acquired immunodeficiency syndrome (AIDS).

In terms of postabortion infectious complications, Stuart and colleagues found that postoperative infection did not differ between women with and without HIV.^72^ A Cochrane review from 2018 did not find any studies examining the safety and efficacy of procedural abortion among individuals living with HIV, emphasizing the lack of evidence to guide health care providers pertaining to this clinical situation.^66^ Overall, these studies do not demonstrate strong evidence for a heightened risk of infection after suction curettage among people living with HIV. However, given the findings in Stuart and colleagues’ small study about the potential increased risk of anesthetic complications among those living with HIV, clinicians should engage in shared decision-making with patients regarding risks and benefits of undergoing abortion care in the outpatient versus operating room setting, particularly for those with HIV without viral suppression or with AIDS.

### FUTURE CONSIDERATIONS

Future research should investigate the drug–drug interactions and pharmacokinetics between hormonal contraception and new ART agents, as well as between medication abortion regimens and HIV drugs. Moreover, as uptake of PrEP for HIV is expected to increase in the coming years, studies should focus on ART agents used for PrEP and their interactions with contraceptive drugs. Another area of research that could provide valuable clinical guidance would be to explore optimal pain management regimens for both medication and procedural abortion among women living with HIV, which remains an underexplored but crucial issue to address in this population. Likewise, further studies should evaluate optimal protocols to minimize infection risk and anesthetic complications for those living with HIV who are undergoing procedural abortion.

## CONCLUSION

There is an unmet need for family planning services for women living with HIV. Given the lack of comprehensive clinical data, professional societies and expert panels have released few validated tools to guide clinicians in providing contraception and abortion care in this population. However, although they are not exhaustive, various pharmacokinetic and clinical studies may help guide patient counseling and shared decision-making. As data are continually evolving in this area, reproductive health care providers should regularly refer to the Centers for Disease Control and Preventions *US Medical Eligibility Criteria for Contraceptive Use* and the US Department of Health and Human Services’ *Guidelines for the Use of Antiretroviral Agents in Adults and Adolescents with HIV* for updated recommendations on specific clinical scenarios. In addition to these guidelines, the Liverpool Drug Interactions resource is a website that lists updated and evidence-based drug–drug interactions with antiretroviral medications.^21^ Providers should always consider undertaking an updated literature search when counseling patients about drug interactions. Conversations regarding risks and benefits of contraception or abortion care should always incorporate similar discussions of the risks and benefits of pregnancy. Eliminating structural barriers to contraception, abortion, and pregnancy care and advocating for patient empowerment are key for reproductive health care providers to help ensure equitable health care access for this population.

## Figures and Tables

**Table 1.** Recommendations for Contraceptive Method Among People Living with HIV Based on Antiretroviral Regimen

Antiretroviral Drug	Cu-IUD		LNG-IUD		Implants	DMPA	POC	CHCs
	
	Initiation	Continuation	Initiation	Continuation				

**Nucleoside reverse transcriptase inhibitors (NRTIs)**								
Tenofovir (TDF)	1/2^a^	1	1/2	1	1	1	1	1
Emtricitabine (FTC)	1/2	1	1/2	1	1	1	1	1
Abacavir (ABC)	1/2	1	1/2	1	1	1	1	1
**Integrase strand inhibitors (INSTIs)**								
Dolutegravir (DTG)	1/2	1	1/2	1	1	1	1	1
Raltegravir (RAL)	1/2	1	1/2	1	1	1	1	1
Elvitegravir (EVG)	1/2	1	1/2	1	1	1	1	1
**Nonnucleoside reverse transcriptase inhibitors (NNRTIs)**								
Efavirenz (EFV)	1/2	1	1/2	1	2	1	2	2
Nevirapine (NVP)	1/2	1	1/2	1	1	1	1	1
Rilpivirine (RPV)	1/2	1	1/2	1	1	1	1	1
**Ritonavir-boosted protease inhibitors**								
Ritonavir-boosted atazanavir (ATV/r)	1/2	1	1/2	1	2	1	2	2
Ritonavir-boosted darunavir (DRV/r)	1/2	1	1/2	1	2	1	2	2
Ritonavir-boosted fosamprenavir (FPV/r)	1/2	1	1/2	1	2	1	2	2
Ritonavir-boosted lopinavir (LPV/r)	1/2	1	1/2	1	1	1	1	1
**Unboosted protease inhibitors (without ritonavir)**								
Atazanavir (ATV)	1/2	1	1/2	1	1	1	1	2
Fosamprenavir (FPV)	1/2	1	1/2	1	2	2	2	3
Indinavir (IDV)	1/2	1	1/2	1	1	1	1	1
Nelfinavir (NFV)	1/2	1	1/2	1	2	1	2	2

Abbreviations: CHC, combined hormonal contraceptive; Cu-IUD, copper-containing intrauterine device; DMPA, depot-medroxyprogesterone acetate; LNG-IUD, levonorgestrel-releasing intrauterine device; POC, progestin-only contraceptive.

a Category definitions, adapted from the *US Medical Eligibility for Contraceptive Use* (2016): 1, A condition for which there is no restriction for the use of the contraceptive method. 2, A condition for which the advantages of using the method generally outweigh the theoretical or proven risks. 3, A condition for which the theoretical or proven risks usually outweigh the advantages of using the method. 4, A condition that represents an unacceptable health risk if the contraceptive method is used. For intrauterine devices, insertion is categorized as category 2 if the person is not treated and clinically unwell. Otherwise, both insertion and continuation are classified as category 1

Source: Adapted from *Summary of Changes from US MEC* (2016).^20^

## Quick Points

- Each clinician should take an individualized approach to contraception counseling among people living with HIV and work closely with the patient’s infectious disease/HIV care provider.
- Resources regarding safety, efficacy, and drug–drug interactions of contraception and antiretroviral therapy are accessible to reproductive health, primary, and infectious disease care providers.
- Although abortion provides benefit for people living with HIV who do not desire to continue pregnancy, data are limited regarding the clinical safety of medication and procedural abortions among people living with HIV, and further research is needed in this area.
